# Supplementary material for: LIPG signaling promotes tumor initiation and metastasis of human basal-like triple-negative breast cancer
Source: eLife. 2018 Jan 19;7:e31334. doi: 10.7554/eLife.31334 (PMC5809145; doi:10.7554/eLife.31334)
Supplement: Transparent reporting form [file elife-31334-transrepform.docx]

***eLife’s* transparent reporting form**

We encourage authors to provide detailed information *within their submission* to facilitate the interpretation and replication of experiments. Authors can upload supporting documentation to indicate the use of appropriate reporting guidelines for health-related research (see [EQUATOR Network](http://www.equator-network.org/%20)), life science research (see the [BioSharing Information Resource](https://biosharing.org/)), or the [ARRIVE guidelines](http://www.plosbiology.org/article/info:doi/10.1371/journal.pbio.1000412) for reporting work involving animal research. Where applicable, authors should refer to any relevant reporting standards documents in this form.

If you have any questions, please consult our Journal Policies and/or contact us: [editorial@elifesciences.org](mailto:editorial@elifesciences.org).

**Sample-size estimation**

- You should state whether an appropriate sample size was computed when the study was being designed
- You should state the statistical method of sample size computation and any required assumptions
- If no explicit power analysis was used, you should describe how you decided what sample (replicate) size (number) to use

Please outline where this information can be found within the submission (e.g., sections or figure legends), or explain why this information doesn’t apply to your submission:

1. For *in silico* analysis of gene expression in Oncomine breast cancer gene expression datasets, the sample sizes of tissue groups were determined by the analyzed datasets.

2. For qRT-PCR and IHC analyses of tissue samples, the sample sizes were determined by the available number of tissue samples in the study.

3. For analysis of xenograft tumor growth curves, six replicates are the minimal sample size for xenograft studies.

4. Five replicates are the minimal sample size for in vivo metastasis studies.

5. For other data, three replicates are the minimal sample size for general experiments.

**Replicates**

- You should report how often each experiment was performed
- You should include a definition of biological versus technical replication
- The data obtained should be provided and sufficient information should be provided to indicate the number of independent biological and/or technical replicates
- If you encountered any outliers, you should describe how these were handled
- Criteria for exclusion/inclusion of data should be clearly stated
- High-throughput sequence data should be uploaded before submission, with a private link for reviewers provided (these are available from both GEO and ArrayExpress)

Please outline where this information can be found within the submission (e.g., sections or figure legends), or explain why this information doesn’t apply to your submission:

1. In our studies, three independent experiments were performed for each designed experiment for statistical analysis. For in vivo animal studies, five or six replicates were performed.

2. In our studies, biological replication is defined as experimental replication for an experiment involving live cells and organisms (e.g cell transfection experiments), and technical replication is defined as experimental replication for an assay (e.g. qRT-PCR). The information with regard to biological and technical replication has been described in figure legends.

3. The definition of the outliers shown in the gene expression datasets has been clearly described in figure legends.

**Statistical reporting**

- Statistical analysis methods should be described and justified
- Raw data should be presented in figures whenever informative to do so (typically when N per group is less than 10)
- For each experiment, you should identify the statistical tests used, exact values of N, definitions of center, methods of multiple test correction, and dispersion and precision measures (e.g., mean, median, SD, SEM, confidence intervals; and, for the major substantive results, a measure of effect size (e.g., Pearson's r, Cohen's d)
- Report exact p-values wherever possible alongside the summary statistics and 95% confidence intervals. These should be reported for all key questions and not only when the p-value is less than 0.05.

Please outline where this information can be found within the submission (e.g., sections or figure legends), or explain why this information doesn’t apply to your submission:

1. F-test was used to analyze variances between two gene expression datasets from the Oncomine database.

2. For qRT-PCR and IHC data analyses, expression differences between tissue groups were analyzed by *t* test for continuous variables.

3. Statistical analysis of tumor growth curves was performed by Two-way ANOVA.

4. Correlation between mRNA levels of two genes was analyzed by Pearson correlation test.

5. Statistical analysis of general experimental datasets was performed by *t* test.

6. The aforementioned statistical methods have been described in the "Materials and Methods" section.

7. Raw data have been presented in figures when N per group is less than 10.

8. The *p*-values are reported in figure legends as * *p* < 0.05; ** *p* < 0.01; *** *p* < 0.001.

(For large datasets, or papers with a very large number of statistical tests, you may upload a single table file with tests, Ns, etc., with reference to sections in the manuscript.)

**Group allocation**

- Indicate how samples were allocated into experimental groups (in the case of clinical studies, please specify allocation to treatment method); if randomization was used, please also state if restricted randomization was applied
- Indicate if masking was used during group allocation, data collection and/or data analysis

Please outline where this information can be found within the submission (e.g., sections or figure legends), or explain why this information doesn’t apply to your submission:

1. For *in silico* data analysis, the expression value for each tissue sample was allocated into an analysis group based on the molecular subtype of the tissue sample.

2. For qRT-PCR and IHC analyses of tissue samples, samples were allocated into experimental groups according to their molecular subtypes.

**Additional data files (“source data”)**

- We encourage you to upload relevant additional data files, such as numerical data that are represented as a graph in a figure, or as a summary table
- Where provided, these should be in the most useful format, and they can be uploaded as “Source data” files linked to a main figure or table
- Include model definition files including the full list of parameters used
- Include code used for data analysis (e.g., R, MatLab)
- Avoid stating that data files are “available upon request”

Please indicate the figures or tables for which source data files have been provided:

All of numerical data that are represented as graphs in figures have been provided as the source data (Excel files) in the submission.
